# A New Ammonium Smart Sensor with Interference Rejection

**DOI:** 10.3390/s20247102

**Published:** 2020-12-11

**Authors:** Juan V. Capella, Alberto Bonastre, José C. Campelo, Rafael Ors, Miguel Peris

**Affiliations:** 1 Instituto de las Tecnologías de la Información y Comunicaciones ITACA, Universitat Politècnica de València, Camino de Vera s/n, 46022 Valencia, Spain; jcapella@itaca.upv.es (J.V.C.); bonastre@itaca.upv.es (A.B.); jcampelo@itaca.upv.es (J.C.C.); rors@itaca.upv.es (R.O.); 2 Department of Chemistry, Universitat Politècnica de València, 46071 Valencia, Spain

**Keywords:** smart ammonium sensor, in-line water monitoring, wireless sensor networks, interference tolerance, expert system, triple modular redundancy

## Abstract

In many water samples, it is important to determine the ammonium concentration in order to obtain an overall picture of the environmental impact of pollutants and human actions, as well as to detect the stage of eutrophization. Ion selective electrodes (ISEs) have been commonly utilized for this purpose, although the presence of interfering ions (potassium and sodium in the case of NH_4_^+^-ISE) represents a handicap in terms of the measurement quality. Furthermore, random malfunctions may give rise to incorrect measurements. Bearing all of that in mind, a smart ammonium sensor with enhanced features has been developed and tested in water samples, as demonstrated and commented on in detail following the presentation of the complete set of experimental measurements that have been successfully carried out. This has been achieved through the implementation of an expert system that supervises a set of ISEs in order to (a) avoid random failures and (b) reject interferences. Our approach may also be suitable for in-line monitoring of the water quality through the implementation of wireless sensor networks.

## 1. Introduction

It is well-known that the presence of NH_4_^+^ ions in groundwater is not a serious health problem, even though high amounts of this cation often indicate water contamination. The content of NH_4_^+^ in unpolluted groundwater is usually under 0.2 mg L^−1^, but can be higher in zones with intensive animal production and in cultivated areas. In fact, ammonium ions in water mainly come from (a) farming; (b) dissimilatory nitrate reduction to ammonium under anaerobic conditions; and (c) anthropogenic sources, such as fertilizers or the leakage of waste water. Both the World Health Organization (WHO) and the European Union have reported the maximum concentrations allowed for ammonium as a function of the type of water [1]. Since the presence of high levels of this ion in water presents a certain risk to health, there is no doubt that monitoring of the ammonium concentration in rivers and aquifers may be of great importance. Several instrumental techniques have been employed for this purpose [2,3], most notably ion selective electrodes (ISEs) due to their effectiveness if we think in terms of in-line monitoring [4,5]. These devices have several important advantages, including a high selectivity and sensitivity, a low cost and energy consumption, ease of use and portability, and a good precision. Furthermore, it should be noted that they perform non-destructive analysis, making them highly appropriate for wireless sensor network (WSN) applications.

Nevertheless, a major drawback in the use of ion selective electrodes is the effect of interfering ions in the water samples to be analyzed [6]. Several approaches with a view to overcoming this difficulty have been proposed based on the combined use of several ISEs, such as the development of electronic tongues, which are arrays of potentiometric sensors (ion selective electrodes) combined with pattern recognition tools. Their performance in water quality monitoring [7,8] has been contrasted with the case of using discrete ISEs [9], sometimes even supported by artificial neural network (ANN) architectures [10]. However, all of these contributions—though successful—are not suitable for WSN applications due to their complexity (high-level computer resources are required) and time consumption. Therefore, simpler approaches, i.e., the alternative of using conventional ion selective electrodes, are usually considered [11]. Our proposal—described in the following pages—is clearly situated in between these approaches; it certainly represents a step forward in the development and utilization of traditional ion selective electrodes, but without needing to resort to other more sophisticated electronic devices.

It is assumed that no ISEs are fully ion-specific [12], although the degree of interference depends on a great deal of factors, thus preventing—in principle—a suitable correction of readings. In general terms, the selectivity coefficient, *k_ij_*, is used to express the intensity of the interference according to the Nicolsky–Eisenman Equation (1) (derived from the Nernst equation) [13]:

(1) $$E_{i}=E_{i}^{0}+2.303\frac{RT}{n_{i}F}log\left\{a_{i}+\sum_{j}k_{ij}\left(a_{j}^{\left(n_{i}/n_{j}\right)}\right)\right\},$$

where *E* is the emf, *E*^0^ is the standard electrode potential, *n* is the ionic valency including the sign, *a* is the activity, *i* is the ion of interest (target ion), *j* represents the interfering ions, and *k_ij_* is the selectivity coefficient. The smaller this value, the smaller the interference by *j* [14]. On the other hand, the concentration of target ion (or more accurately, the ratio of target ion to the interfering ion concentration) is inversely proportional to the degree of interference

The ideal ion selective electrode should be interference-free [15], although what most manufacturers try to achieve is to keep these interferences to a minimum. Nevertheless, recent progress in the field of communications and microelectronics (including data management) permits technologically advanced systems to be obtained [16]. In fact, Capella and co-workers addressed this issue in the determination of nitrates [15] and reached a satisfactory solution, with interferences being eliminated. Nevertheless, this solution did not consider the possibility of ISE malfunctions and their errors, be they transient (incorrect measurements at random) or permanent (drifting in the electrodes). Then, and as a result of the experience accumulated on the employment of these techniques, we observed that additional work was required to deal with these failures. Therefore, our proposal, which is presented in this article, involves new strategies to minimize their effects. 

In our case, the cornerstone has been the utilization of the triple modular redundancy (TMR) technique [17], as well as a rule-based expert system [18]. In the TMR technique, each measurement is taken by three devices, and the average value is calculated, as well as the corresponding deviations. The interference rejection mechanism makes use of additional ISEs (also in threes for the aforementioned reason) to evaluate the presence of interfering ions in the water sample. In this sense, we have developed an ammonium smart sensor that can remove the main interferences from other ions, namely potassium [19] and sodium (as justified in Section 3.2.1). Furthermore, it meets the major requirements for being employed in a WSN-based application [20], with all the corresponding benefits in the field of analytical chemistry. 

Based on the determined concentration of interferents and the [NH_4_^+^] obtained, a correction line is applied to estimate the real value of the ammonium concentration. This line is found by means of a linear regression of the data provided by a set of experiments. 

The expert system utilized is based on rules, and follows the so-called Rule Nets mechanisms [18]. This allows the system behavior to be carefully detailed in a simple, swift way. The knowledge of chemical experts is then transferred to the computer control. In our case, the implemented rules only refer to TMR management and interference rejection.

### 1.1. Interference Rejection

As stated above, the presence of interferences is a serious drawback in the determination of a chemical species by means of ion selective electrodes. In order to reduce the effect of these interfering agents, a correction mechanism has been proposed that permits an estimation of the analyte concentration to be obtained by offsetting the interferences from other species. Nevertheless, the complexity of this approach should be noted, since the determination of the concentration of interferents is also affected by other species (including the analyte). 

The starting point of the interference rejection technique proposed is the hypothesis that the real measurement is a linear combination of the values obtained by the ISEs of ammonium, sodium, and potassium, in such a way that it is possible to estimate—using a linear regression—the corresponding coefficients, and then the concentration of NH_4_^+^ as a function of the ISE measurements, in the following way:

[NH_4_^+^]_estimated_ = m_1_ × [NH_4_^+^]_measured_ + m_2_ × [K^+^]_measured_ + m_3_ × [Na^+^]_measured_ + b. (2)

The calculation of the aforementioned coefficients is carried out after the corresponding experiments.

### 1.2. Transducer Redundancy

As previously indicated, a key objective in the design of new sensors suitable for WSNs is to offer a high dependability (the degree to which the result of a measurement, calculation, or specification can be depended on to be accurate). To achieve this goal, several techniques can be utilized, mostly depending on the sensor’s working environment. The following aspects should then be taken into account:

1. Within the structure of a mote, the reliability of the electronic components, such as the microcontroller, which are often encapsulated in waterproof industrial enclosures, is between 100 and 1000 times higher than that of the ISEs.

2. Being increasingly exposed to aggressive media, ISEs are more susceptible to failures, be they random [21] or permanent.

Therefore, the reliability of the proposed system will mostly depend on the ISE. Its failure will give rise to an anomalous measurement, and subsequently, to erroneous decisions/actions taken from that measurement. Fortunately, sensors are passive, i.e., they only pass their information to the microcontroller they are connected to. This enables the option of using many sensors to mitigate several types of failures, thus improving the reliability of the system. 

Assuming that ISEs are the less reliable elements of our system, we propose recourse to a triple modular redundant (TMR) structure for these measuring elements. In this way, three transducers will be employed (instead of just one) for each of the magnitudes measured. 

A TMR system is a particular case of N-modular redundant systems (with N = 3), where N modules usually perform the same action, and the information obtained is then processed so as to select the correct measurement. In this case, failure in one of the ISEs can be tolerated and then,

- Both transient and permanent failures are immediately masked;
- No additional technique is necessary in the ISEs prior to the masking of the failure;
- The conversion of a non-redundant system into a redundant one is not a complex process.

Finally, all transducers must be provided with the necessary intelligence to control their functioning as if they were a single device, but with a higher reliability. This intelligence is provided by the rule-based expert system.

### 1.3. Rule-Based Expert System

Since motes are ultimately computer systems, their software should also be studied. This can be divided into the following:

- Microcontroller system software;
- Communication software (sending and reception of information through the network);
- Application software (this establishes the functioning of the mote from the application’s point of view).

Taking into account the fact that our goal is to increase the dependability of the motes, it is necessary to provide them with a certain degree of intelligence. That is why we have resorted to using artificial intelligence techniques [22], and as an application software, a rule-based expert system has been implemented to control their functioning. 

The reason for this is two-fold:

- On one hand, it is a programming technique that offers a great flexibility for establishing the suitable functioning of the mote, since it must be easily adaptable to other application requirements.
- On the other hand, the system needs to be user-friendly, and knowledge-based systems have enabled unexperienced users to specify the required functioning of the motes by means of simple rules. This option has previously been dealt with by our research group, and an expert system has been developed (by chemists) for the monitoring and control of chemical analysis processes [23].

This article is structured as follows: Section 2 describes the entire system; Section 3 contains a full description of the experimental procedure; and Section 4 shows the results obtained and the corresponding discussion. 

## 2. System Description

The spectacular development of Information and Communication Technologies has led to new paradigms, some of which will very likely be vital in the future of our society. A promising example is the wireless sensor networks (WSN), since they offer a real-time, spatially distributed monitoring system that permits a vast amount of information to be obtained at a reasonable cost. WSNs are based on the deployment of a great number of sensor nodes (called “motes”) that are connected to one another, as well as to a node known as Gateway (in charge of collecting all data from the motes) through a wireless network. Each mote takes measurements using its sensors, and they are then transmitted to a neighboring node, until reaching the Gateway. At this moment, the Gateway stores the information somewhere so that it can be processed and analyzed by the end-user. Nowadays, most gateways are connected to the Internet, which allows for the remote storage of this information. Given the spatial distribution of the sensor nodes and the fact that the information they send reaches the end-user with very low latency times, this user can access real-time information. The storage and further processing of all this information may offer the possibility of studying the evolution of a certain parameter (for example, a pollutant) over time and space. 

WSNs also bring other important advantages (over traditional data acquisition systems), such as a low cost system, high availability of measurements, high system reliability, accessibility, flexibility, scalability, user-friendliness, and size [18]. The dramatic drop in prices of computer systems and the extraordinary simplicity of mote electronics make WSNs rather inexpensive. On the other hand, since the mote may offer different options for selecting times to take measurements, this results in a high availability of measurements. The existence of a great number of motes makes the system very robust against eventual failures in some of them. Moreover, since they are a computer system, their proper functioning is ensured to a much greater extent (as described in Section 2.2). Motes may be easily deployed, even in dangerous or hard-to-access environments, and they can also be relocated in places where a higher data density may be required. The design of WSNs tends to make their use easier; it should also be noted that the term “mote” refers to the very small size of sensor nodes. 

That being said, the technology behind WSNs is neither easy nor problem-free. In the field of chemical analysis, several important challenges need to be solved, for example, the development of new transducers compatible with this technology, i.e., devices with a small size, low price, and low consumption. Due to these limitations, the most commonly used transducers are (a) those based on either fluorescence or UV absorption measurements, (b) ion selective electrodes (ISE), and (c) impedance sensors [24]. 

### 2.1. Wireless Sensor Networks (WSN)

#### 2.1.1. Structure

A WSN consists of three different elements: The motes; the Gateway node; and the Information Management System (IMS).

The motes (sensor nodes) are in charge of obtaining the information to be monitored and sending it to the Gateway. Each mote is composed of the following:

- A microcontroller chip (computer with a low-cost, low-consumption, and low-size integrated circuit). This microcontroller is equipped with a CPU, memory, and different input/output elements. The CPU runs the software found in the memory and interacts with the rest of the system through the corresponding input/output circuits;
- A power supply unit. This is in charge of providing the mote with the energy required for its correct functioning;
- The communications module. This allows the motes—along with the Gateway—to form a network (the WSN). The limiting characteristic of this network is its short length due to the consumption constraints, and therefore, the information needs to be transmitted from node to node until reaching the Gateway;
- The transducers permit the value of a physical magnitude to be determined by indirectly converting the value of the said parameter into electric signals (proportional to that value) that can be processed and transmitted through the microcontroller.

The Gateway node is responsible for receiving the information from the motes and transmitting it to the Information Management System. This transmission is usually carried out by means of the Internet (since it often has a link to a wired/wireless public network).

Finally, the Information Management System (IMS) is the computer system in charge of (a) receiving all data from the Gateway, (b) saving them to the internal memory, and (c) processing this information so that it can be offered in the required format.

#### 2.1.2. Functioning

When an area is to be monitored using a wireless sensor network, the first step is the deployment of the WSN, for which the points where the motes are to be located should be placed on a plane. The density of these points has two extreme values: The lower limit is the minimum distance (short, as mentioned above), whereas the upper limit is governed by both the “granularity” with which data are to be obtained and the economic resources available. Once the motes are properly located, in the second step, motes start to work by establishing the topology of the network. There are many protocols that can be applied for this purpose [25]. The aim consists of looking for a path to the Gateway from each mote. According to a usual procedure, each mote analyzes its ability to directly connect with the Gateway. If this is possible, its information reaches the Gateway in one leap; otherwise, that mote will look for another one already connected, which means two leaps. This continues to occur until all of the motes are able to reach the Gateway in a certain number of leaps, which is why these networks are often called multi-hop networks. The consequence of this is that the topology of the resulting network resembles a tree, where the Gateway is the trunk and the motes lying furthest away are the leaves, in such a way that the further the mote is from the trunk, the more leaps it needs for its information to reach the Gateway. The third (and last) step involves the normal operation mode of the WSN. Motes begin to gather and transmit the information. For this purpose, the microcontroller (following the preset time sequence) takes a measurement by means of the transducer and sends the corresponding data to the node with which it is connected. In this way, the information flows into the Gateway, which receives data from the different motes and “uploads” them on the IMS. Additionally, the Gateway may take on other important tasks related to certain organization and control of the network, initial data processing, and so on. In terms of the communication between the Gateway and the IMS, it should be highlighted that this system is increasingly based on the Internet and its services; in this sense, the current commercial networks (4G, 5G, ADSL, and optical fiber) play a relevant role, along with the substantial increase in the storage and processing in the Cloud [26]. In short, and from the point of view of the IMS, the current evolution is leading from an IMS based on a single server to an entity formed around services in the Cloud (where the information is stored, processed, and made available to the user). 

### 2.2. Smart Sensor Techniques

One of the main goals of this work is to develop techniques resulting in smarter sensors, with an increase in the proper functioning of every mote (a better data acquisition system). The latter will be achieved by using interference rejection procedures and transducer redundancy, since this is a highly sensitive point. With respect to the supervision of the general functioning of the mote, intelligence will be increased by means of a rule-based expert system. It will also monitor the techniques applied to the acquisition. 

**Figure 1:**
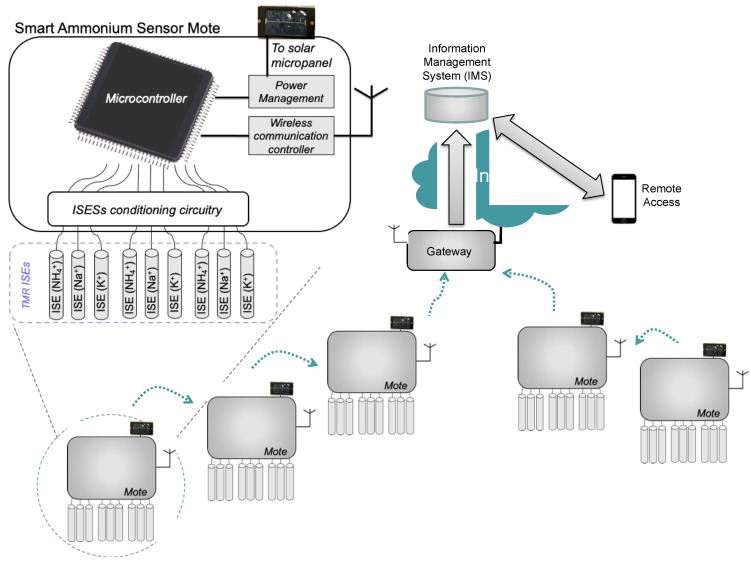
Proposed system architecture.

Figure 1 outlines the architecture of the proposed solution. It highlights the structure of the motes, with the microcontroller that supervises the operation of the sensor subsystem (including the techniques leading to a smart sensor, namely, the rejection of interferences and transducer redundancy). 

In this way, the microcontroller executes the rule-based expert system software. Basically, it consists of a set of *If-Then* rules that permit the coordinated operation of all sensor elements to be controlled. These rules can be divided into three types: (a) Some of them determine how the analyses are carried out; (b) others control the energy and communication issues; and (c) the rest are in charge of increasing the reliability of the system by taking appropriate actions that allow for the sensor to yield a correct response, even if an error occurs. 

The WSN is composed of a set a motes and the communications—as previously explained—occur on a hop-by-hop basis, until reaching the Gateway. This Gateway is connected to the Internet and thus to the IMS. 

## 3. Experiment

### 3.1. Materials and Reagents

Stock solutions of NH_4_Cl (for ammonium), KCl (for potassium), and NaCl (for sodium) were obtained from a suitable amount of the corresponding reagent (analytical grade) using distilled and deionized water (specific conductivity < 0.2 µs cm^−1^). Thereafter, standard solutions were obtained by appropriate dilution.

Ammonium, potassium, and sodium concentrations were measured by means of the corresponding homemade ISEs, fabricated and conditioned according to the bibliography [27]. Their main features are summarized in Table 1. On the other hand, validation of the results obtained by the three ISEs was carried out by utilizing the corresponding official standard methods [28].

In order to perform the measurements, all electrodes were simultaneously immersed in solutions (prepared by appropriate mixtures of the corresponding stock solutions), with the following resulting concentrations: (a) 0.5, 1.0, 1.5, and 2.0 mg L^−1^ of ammonium; (b) 1.0, 3.5, 6.0, and 8.5 mg L^−1^ of potassium; and (c) 20.0, 50.0, 80.0, and 110.0 mg L^−1^ of sodium. The contents of NH_4_^+^, K^+^, and Na^+^ were then measured by combining these solutions in threes, as shown in Table 2.

Our new smart sensor node includes a low-power, inexpensive microcontroller with a sufficient capacity to perform all of the operations required to apply the developed techniques. It consists of a small-size integrated circuit containing all of the computer components (CPU, memory, and necessary I/O subsystems), in such a way that it is possible to implement full applications utilizing a single chip.

Table 3 summarizes the main characteristics of the device chosen [29]. The analog signals from these ISEs are conveniently multiplexed [30], and then processed and amplified by means of analogical circuits. The output of this circuit is received by the A/D converters of the micro-controller (Input/Output subsystems) to be discretized. After suitable conversion has been achieved, the digital information of each ISE measurement is available and, therefore, able to be processed. Finally, the [NH_4_^+^] measured is corrected by means of the interference rejection mechanism, and an estimated [NH_4_^+^] value which will be considered the right measurement is calculated.

After being obtained, the corrected value is transmitted by the communication subsystem, either periodically (upon request) or automatically when the determined conditions are fulfilled [31].

Owing to the fact that one of the objectives was the potential employment of this interference-tolerant ammonium smart sensor node in Internet of Things (IoT) applications, consumption and maintenance should be minimized [32]; that is why low-power devices have been used.

### 3.2. Methods

#### 3.2.1. Rejection of Interferences

As mentioned above, a major problem in the determination of the ammonium concentration by means of ISEs is the presence of potentially interfering ions, since the results obtained may be higher than expected. In our case [19], the corresponding selectivity coefficients (in brackets) show that the following ions usually interfere: K^+^ (10^−1^); Na^+^ (2 × 10^−3^); Mg^2+^ (2 × 10^−4^); Ca^2+^ (6 × 10^−5^); and Li^+^ (3 × 10^−5^). The two interfering species with the highest coefficient values, i.e., potassium and sodium, will thus be considered, since they present the lowest selectivity. However, the procedure proposed in this paper could be applied to other potentially interfering cations, without any difficulty. It should also be noted that the potassium and sodium concentrations have been selected by taking into account the values obtained found in previous field sample determinations. It is also important to highlight that in the area of Electroanalytical Chemistry, it is usually admitted that, when the ionic strength (IS) is lower than 0.01 M for monovalent ions (i.e., very dilute solutions), the activities of the substances in the solution closely approach concentrations, so the utilization of concentration units (and not activity) for the measurements should not lead to a significant error in the results obtained, even without the employment of ionic strength adjustment buffer (ISAB). In the case of the present investigation, ions occurring in the solutions used were all monovalent and the IS of the most concentrated solution was 0.00256 M, which is why concentrations—and not activities—were used throughout the entire work (including calibration).

The estimation of the accumulative error produced in the obtained measurement is the basis of the correction procedure. It has been proven that this error depends on the concentration of the analyte (NH_4_^+^), as well as the interfering ions (K^+^ and Na^+^). Our starting point was thus the assumption that it is feasible to evaluate, and then try to compensate for, the interference error, provided that [K^+^] and [Na^+^] are known. For this purpose, additional potassium and sodium ISEs were incorporated, along with the ammonium ISEs, so as to measure the concentrations of these three ions. It should be taken into account that the real concentrations are not available, since they must be obtained by the measurements of these electrodes, and in all likelihood, they will be influenced by the interfering species (and maybe other instrumental errors). 

In this sense, a very comprehensive set of experiments (shown in Table 2) was then performed under laboratory conditions, in such a way that the nine ISEs were applied to mixtures obtained from known values of [NH_4_^+^], [K^+^], and [Na^+^]. Unsurprisingly, the measured ammonium concentrations exhibited a significant relative error (easily estimated as the difference between measured values and true values). On the other hand, those corresponding to potassium and sodium measurements did not exceed 5% in most instances.

In order to obtain reliable results, *n* replications were carried out for each measurement, with *n* being calculated in the following way. 

The results for each measurement were considered random variables (*X*_1_, *X*_2_, …, *X_n_*) with a *μ* mean value. *n* simulations were repeated until an estimation of *μ* was obtained with a 90% confidence interval according to Equation (3):

(3) X¯(n)±tn−1,0.95S2(n)n,

where *t_n_*_−1,0.95_ is the upper limit of the Student’s *t*-distribution based on *n −* 1 degrees of freedom, *X*(*n*) is the mean value of the results obtained in the different experiments, and *S*^2^(*n*) is the variance of the results obtained in the different measurements.

As a rule, 5–12 replications were performed for each measurement. 

Several techniques were tested to relate the interferences of potassium and sodium to ammonium measurements, from mere corrections to procedures based on artificial intelligence (such as neural networks). From these experiments, it can be observed that the former are not useful in all cases. Instead, the proposed methodology makes it unnecessary to resort to other more complex procedures.

As mentioned above, the starting point is the hypothesis that the real measurement is a linear combination of the values obtained by the ISEs of ammonium, sodium, and potassium, in the following way:

[NH_4_^+^]_estimated_ = m_1_ × [NH_4_^+^]_measured_ + m_2_ × [K^+^]_measured_ + m_3_ × [Na^+^]_measured_ + b. (4)

For sample correlation, linear regression was utilized. After applying the linear regression technique, the corresponding results were obtained, which are shown in Table 4 and Table 5.

The proposed technique is based on a regression analysis (ordinary least squares) that was performed to correlate the values obtained from the three ISEs with the error in ammonium measurements. Linear regression proved to be satisfactory (r^2^ = 0.998986634) and the errors were very low (residual sum of squares = 0.02), which means that the procedure was successful. This accuracy is higher, for instance, than the measurement error. 

With these results, the real ammonium concentration may be estimated by means of a linear expression of measured concentrations of both ammonium and interferences. The coefficients of this linear expression are shown in Table 4.

This procedure also addresses the issue of cross-ion interferences, because the regression line is based on the concentrations measured by the ISEs, instead of on true concentrations. Therefore, mutual interferences were also taken into account.

#### 3.2.2. Transducer Redundancy

As commented on before, a TMR structure is used to improve the reliability of the measuring subsystem. Three ISEs are employed for each chemical species to be determined, namely, NH_4_^+^, K^+^, and Na^+^. 

The processing of the information obtained for each cation is as follows: If all three transducers yield correct measurements, the mean value is considered. In this way, the average error is minimized. Nevertheless (and this clearly shows the advantages of using the TMR technique), if one of the measurements significantly differs from the other two, the malfunctioning of that ISE is suspected and its measurements are discarded, so the mean value of the remaining two is considered. This allows the rejection of random failures (often typical of ISEs), as well as permanent failure of one of the three ISEs [19].

Lastly, if mixed results are obtained with all three ISEs, the system is not able to obtain reliable measurements, which makes the mote communicate its inability to offer this information. This will happen when two or more ISEs of the same species do not work properly; if it is permanent, decision making from the mote’s data will be void. However, in this case, it would be possible to resort to other fault-tolerance techniques; for example, in WSNs, the results of the measurements of a defective mote can be inferred from those obtained by neighboring nodes. 

#### 3.2.3. Ruled-Based Expert System

The expert system (a tuned-up version of a previous one [18]) controlling the functioning of a mote is made up of a set of rules that govern its performance. A rule is a construction with the structure “if (antecedent) then (consequent)”. When all antecedents are met, the rule can be applied, which means that all consequents will be right. Then, one rule may be followed by another one, and so on. Some interesting examples of these types of rules, along with their application (within an expert system), in chemical analysis processes are presented in [33].

In the experimental mote developed in the present work, the rules implemented are in charge of (a) deciding when samples must be taken; (b) indicating how the measurement has to be processed by applying the TMR techniques, as well as the algorithm of interference rejection; (c) controlling the energy consumption; and (d) ensuring error-free functioning of the system. The global performance of all of them (after the corresponding adjustment and setup) permits errors in the measurements to be eliminated, as well as interferences to be rejected, thus resulting in a fault-tolerant smart sensor.

### 3.3. Experimental Description

To demonstrate the applicability of the proposed smart sensor in in-line chemical analysis, a combined experiment that verifies the three aforementioned techniques was carried out in our laboratory to measure the evolution of the ammonium concentration. Special emphasis will be placed on addressing the two main technological issues discussed in the preceding sections (transducers and energy).

First of all, the behavior of the mote was checked when ISE malfunctions occurred. Since this may take some time, we decided to use fault injection techniques [34] to generate intermittent and permanent failures in the laboratory. In both cases, the TMR structure (controlled by the expert system) was able to tolerate the erroneous measurements taken by any of the ISEs, and yielded the correct value.

From the data provided by the TMR (whose reliability is higher than that of a single ISE), the interference rejection technique was evaluated.

Once the correlation coefficients were obtained (following the procedure described in Section 3.2.1), an experiment was proposed to evaluate the quality of the correction. To do this, a set of routines was added (for evaluation purposes) to the expert system, in order to apply the expression (2) with the coefficients of Table 2 on the measurements obtained during the calibration process. 

The results obtained are shown in Section 4.

## 4. Results and Discussion

The software of the expert system applied the previously described error estimation method to the measurements obtained. The calculated error was then subtracted from the [NH_4_^+^] measured to obtain the corrected value. From that moment on, the error was lower than 8% in all instances. Ninety percent of cases displayed errors under 4%, and 67% under 2%. It must be highlighted that all these values are significantly lower than those obtained by the ISEs in the presence of interferences, but without the rejection mechanism. 

**Figure 2:**
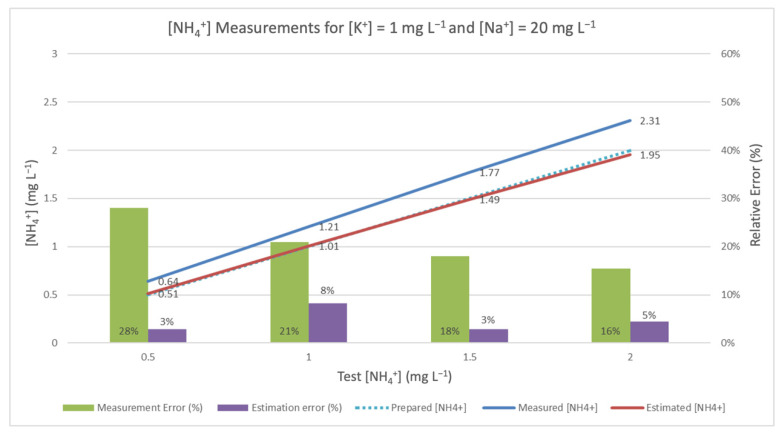
Measured and corrected [NH_4_^+^] in the presence of [K^+^] = 1.0 mg L^−1^ and [Na^+^] = 20 mg L^−1^.

**Figure 3:**
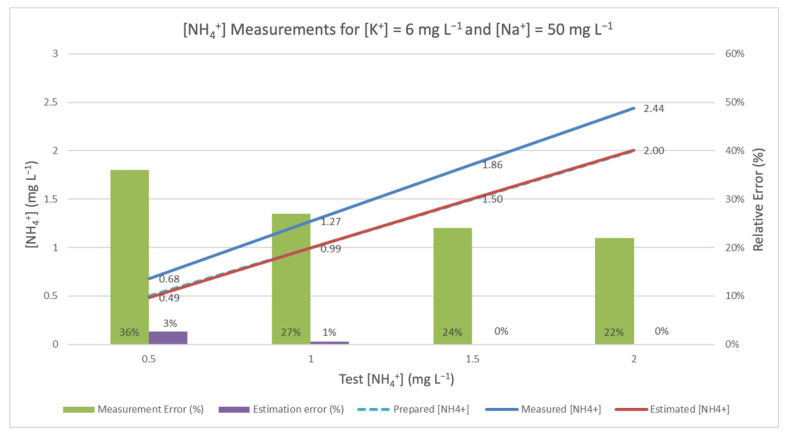
Measured and corrected [NH_4_^+^] in the presence of [K^+^] = 6.0 mg L^−1^ and [Na^+^] = 50 mg L^−1^.

**Figure 4:**
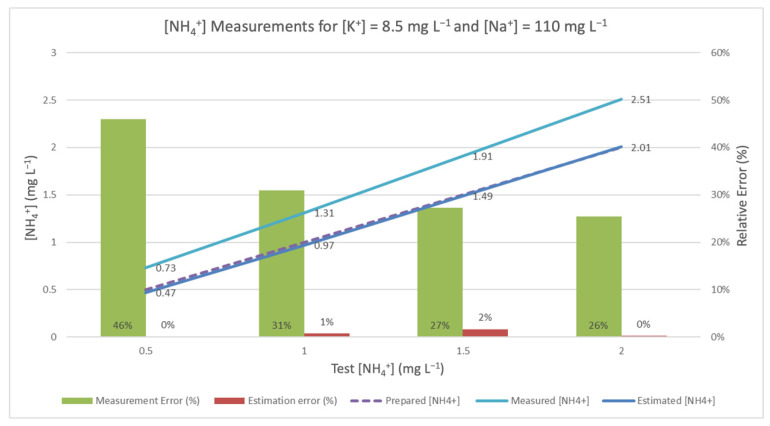
Measured and corrected [NH_4_^+^] in the presence of [K^+^] = 8.5 mg L^−1^ and [Na^+^] = 110 mg L^−1^.

When this correlation was applied to the estimation of NH_4_^+^ measurement, the results were as shown in Figure 2, Figure 3 and Figure 4. They underscore the difference between the measurement obtained by ammonium-ISE and the one estimated using the aforementioned method (for standard solutions of ammonium, potassium, and sodium). In all cases, it should be noted that ISE malfunctions, be they incidental or intentional (see Section 3.3), are responsible for the frequently high relative errors obtained in the absence of TMR and interference rejection techniques.

Figure 2 shows a comparison of the measures obtained from a single [NH_4_^+^] ISE when the electrode is introduced in standard solutions, where [K^+^] = 1 mg L^−1^ and [Na^+^] = 20 mg L^−1^, and [NH_4_^+^] varies from 0.5 to 2 mg L^−1^, in increments of 0.5 mg L^−1^.

[NH_4_^+^] in the sample is presented on the X axis, whereas the obtained measurements (left Y axis) and the relative errors (right Y axis) are also shown. The upper blue line and the related digits show the measurements obtained by the [NH_4_^+^] ISE. The relative error, calculated as the difference between the measured value and true value, divided by the true value, is reflected by the green bar. This error may be considered relevant, as it was above 15% in all cases.

In order to apply the mechanism to reject the interferences, the mote also measures [K^+^] and [Na^+^], whose results may be affected by the interference of [NH_4_^+^]. All three values are used in Equation (2) to calculate an estimated [NH_4_^+^] value. These values are represented by the red line, and the corresponding estimation errors are represented by the purple bar. As a reference, the true [NH_4_^+^] is represented in the figure by the discontinuous blue line. The closer the estimated measure is to this discontinuous line, the better the estimate. In Figure 2, the interference considered is the lowest of all the experiments. Figure 3 shows the results of the experiments with intermediate values of interference, and Figure 4 presents the experiments with the highest concentrations of K^+^ and Na^+^.

**Figure 5:**
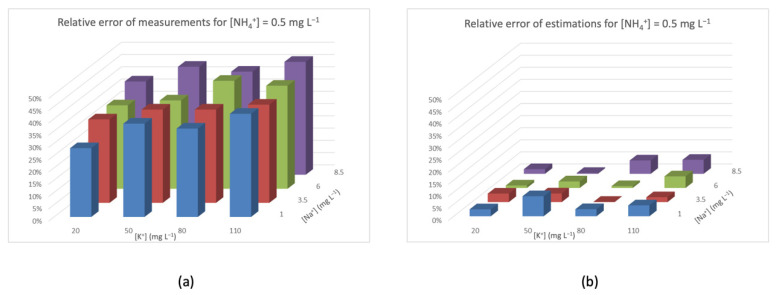
Relative error for [NH_4_^+^] = 0.5 mg L^−1^ (**a**) measured by the ISE and (**b**) after triple modular redundancy (TMR) and interference rejection.

**Figure 6:**
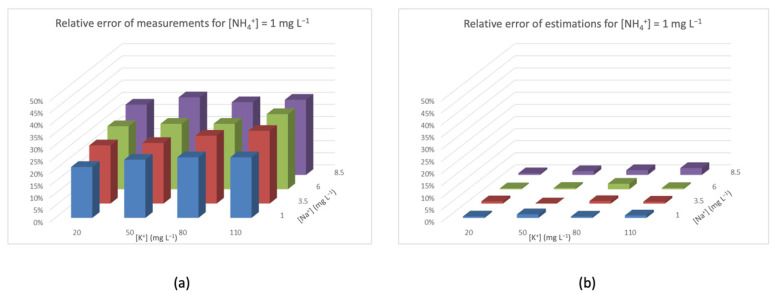
Relative error for [NH_4_^+^] = 1 mg L^−1^ (**a**) measured by the ISE and (**b**) after TMR and interference rejection.

**Figure 7:**
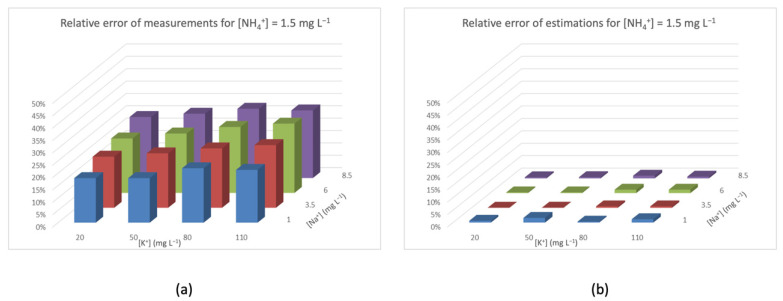
Relative error for [NH_4_^+^] = 1.5 mg L^−1^ (**a**) measured by the ISE and (**b**) after TMR and interference rejection.

**Figure 8:**
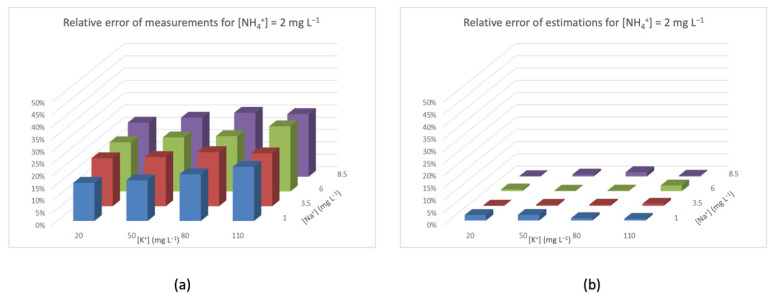
Relative error for [NH_4_^+^] = 2 mg L^−1^ (**a**) measured by the ISE and (**b**) after TMR and interference rejection.

Figure 5, Figure 6, Figure 7 and Figure 8 represent the distribution of relative error (*Z*-axis) with a fixed concentration of ammonium and different concentrations of interfering species ([K^+^] on the *X*-axis, and [Na^+^] on the *Y*-axis). The different colors of the columns identify the concentration of [Na^+^]. In all of these figures, graph (a) shows the relative error of the measures obtained with the NH_4_^+^-ISEs, and graph (b) shows the relative error after applying the aforementioned interference rejection estimation. In Figure 5a, the concentration of NH_4_^+^ is low, so the interference-caused error is added to the relative error of the ISE. It can also be seen that the relative error increases with the concentration of interferences, reaching almost 45% at the highest values. On the other hand, in Figure 5b, the relative errors are much lower, and also irregular because of the relative error of the ISE device.

On the other hand, in Figure 8a, the measured [NH_4_^+^] is high, so the discretization error of the ISE is less relevant. The relative error varies from 10% to 25%. Furthermore, corrected measures also benefit from the lower importance of the discretization error, and are bounded in a 0–3% range.

Clearly, the application of this correction method allows for a significant improvement of the quality of ISE determinations, by drastically reducing the relative errors obtained. A further decrease is not possible due to the precision of the ISEs, but because of this fact, it can be observed that the higher the values of [NH_4_^+^], the lower the relative errors.

## 5. Conclusions

This study presents a new smart sensor with fault tolerance and interference rejection based on simple ion selective electrodes (ISEs) and an expert system. For this purpose, three different techniques have been used: (i) A triple modular-redundant ISE structure, to avoid —among other issues—indeterminate errors; (ii) a procedure based on interfering ion concentration measurement, error estimation, and a correction method; and iii) a knowledge-based system, which supervises and ensures the correct functioning of the whole mote.

The procedure described was applied to measurements obtained in water samples by an NH_4_^+^-ISE in the presence of potassium and sodium (the two most important interfering ions). The smart sensor node developed consists of the nine ISEs utilized (3 × NH_4_^+^, 3 × K^+^, and 3 × Na^+^) and a suitable, inexpensive microcontroller. This ISE replication (in a TMR structure) was carried out to improve the measurement reliability. The microcontroller is then in charge of receiving and processing the values of the measurements obtained.

The results obtained are very promising, with measurement errors being substantially reduced. This is mainly due to the TMR structure (much lower random failures) and the interference rejection mechanism (decreased influence of interfering ions on the measurement obtained). The final outcome after the application of the suggested procedure is that the ammonium measurements provided by this smart sensor are nearly free of interferences.

Lastly, the importance of this approach derives from the application of automatic methods implemented on microcontrollers to offset physical shortcomings, for example, the influence of interferences, in measurement processes. In this sense, the latest generation smart sensors based on these elements may be of great utility for integration within WSNs, on which IoT (Internet of Things) and Ambient Intelligence will rest in the years to come.

## Figures and Tables

**Table 1:** Characteristics of the three ion selective electrodes (ISEs) used.

ISE	Membrane Preparation	Calibration	Correlation with the Official Method (*r^2^* Value)	Nernstian Slope (mV Per Decade Change in Activity)	Limit of Detection (mg L^−1^ of Analyte)	Response Time (s)	Drift (mV h^−1^) in 0.01 M Standard Solutions
NH_4_^+^	400 mg of a mixture of 31% carboxylated polyvinylchloride, 4% nonactin, and 65% bis-(2-ethyl)hexyl sebacate in 5 mL of tetrahydrofurane (THF). Inner electrode solution: 10^−2^ M NH_4_Cl	NH_4_Cl standard solutions	0.9937 in the range 0.1–5.0 mg L^−1^	54 ± 5	0.02	<5	<0.4
K^+^	Tetrahydrofuran solution of 4% benzo-15-crown-5 crown ether, 28% polyvinyl chloride, and 68% orto-nitrophenylphenylether (plasticizer). Inner electrode solution: 0.01 M KCl	KCl standard solutions	0.9947 in the range 0.5–20.0 mg L^−1^	55 ± 4	0.4	<10	<0.5
Na^+^	4% ionophore, 65% dioctylphthalate, 30% polyvinyl chloride (PVC), and 1% potassium tetrakis(*p*-chlorophenyl)borate, in tetrahydrofurane (THF)	NaCl standard solutions	0.9972 in the range 1.0–200.0 mg L^−1^	55 ± 5	0.5	<10	<0.4

**Table 2:** Design of the experiment.

		[K^+^] (mg L^−1^)			
		1.0	3.5	6.0	8.5
[Na^+^] (mg L^−1^)	20.0	[NH_4_^+^] (mg L^−1^) = {0.5, 1.0, 1.5, 2.0}			
	50.0				
	80.0				
	110.0				

**Table 3:** ARM Cortex M4 microcontroller features.

	STM32L422xx
SRAM memory	40 KB
Flash memory	128 KB
Performance	1.25 DMIPS/MHz (Drystone 2.1)
AD converters	2 × 12-bit
Integrated timers	10 (16 and 32 -bit)

**Table 4:** Results of the linear regression.

	ISE Na^+^ (m_3_)	ISE K^+^ (m_2_)	ISE NH_4_^+^ (m_1_)	Term Independent (b)
Coefficients	−5.66975 × 10^−4^	−9.06103 × 10^−3^	8.62656 × 10^−1^	−1.73289 × 10^−2^
Regression standard error	6. 75259 × 10^−5^	8.20620 × 10^−4^	3.54723 × 10^−3^	8.15947 × 10^−3^

**Table 5:** Linear regression parameters.

Correlation Coefficient (r^2^)	9.98986 ×10^−1^	Standard Error	1.83790 × 10^−2^
F value	1.971619 × 10^4^	Degrees of freedom	60
Regression squared sum	1.99797 × 10^1^	Residual sum of squares	2.02673 × 10^−2^
